# Frequency-specific adaptation and its underlying circuit model in the auditory midbrain

**DOI:** 10.3389/fncir.2015.00055

**Published:** 2015-10-01

**Authors:** Li Shen, Lingyun Zhao, Bo Hong

**Affiliations:** Department of Biomedical Engineering, School of Medicine, Tsinghua UniversityBeijing, China

**Keywords:** stimulus-specific adaptation, spectral receptive field, inferior colliculus, center-surround, feedforward model

## Abstract

Receptive fields of sensory neurons are considered to be dynamic and depend on the stimulus history. In the auditory system, evidence of dynamic frequency-receptive fields has been found following stimulus-specific adaptation (SSA). However, the underlying mechanism and circuitry of SSA have not been fully elucidated. Here, we studied how frequency-receptive fields of neurons in rat inferior colliculus (IC) changed when exposed to a biased tone sequence. Pure tone with one specific frequency (adaptor) was presented markedly more often than others. The adapted tuning was compared with the original tuning measured with an unbiased sequence. We found inhomogeneous changes in frequency tuning in IC, exhibiting a center-surround pattern with respect to the neuron's best frequency. Central adaptors elicited strong suppressive and repulsive changes while flank adaptors induced facilitative and attractive changes. Moreover, we proposed a two-layer model of the underlying network, which not only reproduced the adaptive changes in the receptive fields but also predicted novelty responses to oddball sequences. These results suggest that frequency-specific adaptation in auditory midbrain can be accounted for by an adapted frequency channel and its lateral spreading of adaptation, which shed light on the organization of the underlying circuitry.

## Introduction

From the perspective of sensory coding, neurons encode information more effectively by adjusting their dynamic range and magnitude of sensitivity with the ongoing stimulus stream (Müller et al., 1999; Brenner et al., 2000; Dragoi et al., 2002; Krekelberg et al., 2006; Sharpee et al., 2006; Gutnisky and Dragoi, 2008; Zhao and Zhaoping, 2011; Benucci et al., 2013). The receptive fields (RFs) of sensory neurons are typically found to vary dynamically (Condon and Weinberger, 1991; Dragoi et al., 2000; Froemke et al., 2007; Lesica and Grothe, 2008; Peng et al., 2010; Froemke and Martins, 2011; Yin et al., 2014). In the visual cortex, a repulsive shift in orientation tuning was observed following repeated exposure to one stimulus orientation (Dragoi et al., 2000), whereas adaptation to a near-preferred direction caused the direction tuning to shift toward the adapted direction in the middle temporal area (Kohn and Movshon, 2004).

In the auditory system, stimulus-specific adaptation (SSA), in which rare stimuli elicit stronger responses than common ones, was first observed by presenting an oddball stimulus sequence with an unbalanced presentation probability of a rare and a common stimulus (Ulanovsky et al., 2003). This phenomenon was then found in both cortical (Ulanovsky et al., 2003, 2004) and sub-cortical areas (Pérez-González et al., 2005; Anderson et al., 2009; Malmierca et al., 2009; Antunes et al., 2010; Zhao et al., 2011). Although the basic properties of SSA in the inferior colliculus (IC), such as its dependence on frequency separation, repetition rate and stimulus probability, have been examined (Zhao et al., 2011; Duque et al., 2012; Pérez-González et al., 2012; Pérez-González and Malmierca, 2012; Anderson and Malmierca, 2013; Ayala et al., 2013), its underlying mechanism and plausible neural circuitry have yet to be elucidated. SSA in the IC was not abolished by deactivation of the primary auditory cortex (Anderson and Malmierca, 2013), suggesting that SSA may originate from the local circuitry in the IC and/or earlier nuclei. We hypothesize that SSA in the auditory midbrain is generated from re-weighting of frequency-specific afferents through dynamic neural circuits (Ayala and Malmierca, 2013; Nelken, 2014). If that is the case, the weakened response to common stimuli will not be limited to the particular stimuli presented but will also occur with tones at neighboring frequencies.

In this study, we measured the frequency tuning (frequency RF) of individual neurons when they were exposed to fast repetition of one frequency stimulus. Unlike previous studies using only two frequencies (one as rare, the other as common), we used one fixed frequency as the adaptor and multiple frequencies around it to probe the RF change during adaptation. Our data showed that adaptors near the best frequency (BF) which is the frequency that elicits the strongest firing in iso-intensity frequency tuning, caused frequency-specific suppression near the adaptor and led to a repulsive shift of the BF, whereas flank adaptors induced a small or opposite effect. A two-layer feedforward model was further proposed to explain the observed RF dynamics. The fitted model predicted the SSA in the IC and suggested a plausible circuitry with an adapted frequency channel and its weighted lateral spreading of adaptation.

## Materials and methods

### Electrophysiological recording

Healthy adult male Sprague-Dawley rats (body weight 200–300 g) were prepared for electrophysiological recording. Animal care and all experimental procedures conformed to the guidelines of, and were approved by the Institutional Animal Care and Use Committee of Tsinghua University. A complete description of similar experimental procedures can be found in our previous study (Zhao et al., 2011). Briefly, rats were anesthetized with urethane (1.4 g/kg, 20% solution, i.p.) before surgery following an injection of atropine sulfate (0.5 mg/kg, i.p). The anesthetic state was monitored based on breathing patterns and the palpebral reflex and was maintained throughout the experiment with supplementary injections of urethane (1/3 of the initial dose, i.p.) if necessary. Medical oxygen (99.5% O_2_) was delivered to the rat through a customized facemask. Body temperature was monitored and maintained at 38°C ± 1°C by a heating blanket (FHC, Bowdoin, ME, USA).

**Figure 1:**
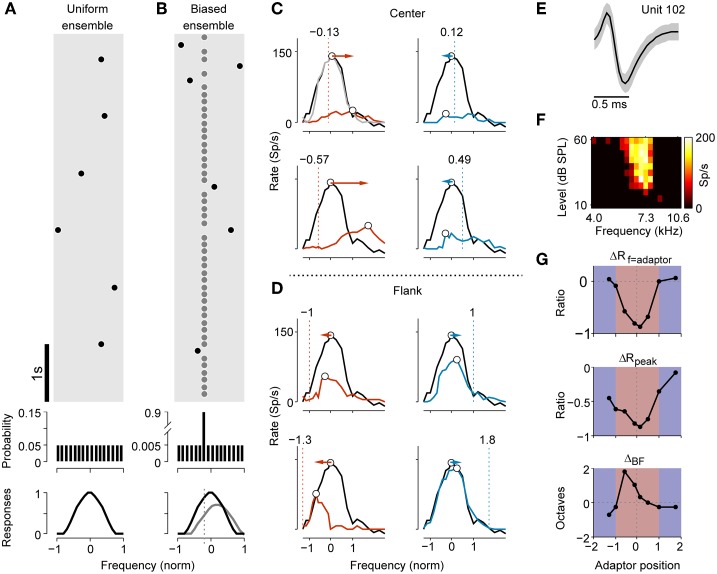
**Stimulus paradigm and adaptive change of frequency tuning in an example neuron. (A)** A segment of the stimulus sequence in the uniform ensemble. Each dot symbolizes a tone. In the entire sequence, the probability distribution of presentation across frequencies was flat (middle panel). The non-adapted frequency-tuning curve to the sequence is illustrated in the bottom panel. **(B)** Stimulus sequence for a biased stimulus ensemble. The frequency probes (black dots) were selected in the same way as in the uniform ensemble, except that they were randomly interspersed in a repeating frequency adaptor (gray dots). The presentation probability of the adaptor was 90%. The bottom panel displays a hypothetic tuning curve for both the original (black) and adapted case (gray). **(C)** Comparison of non-adapted (black) and adapted tuning (colored) with adaptors close to the original BF (center adaptor) of an example neuron. The dashed lines and texts above annotate the normalized frequency of the adaptor (adaptor position). The frequency of the adaptor was below (red) or above (blue) the original BF. The light gray curve in the top left panel shows a second measurement of the non-adapted tuning after 30 s of recovery. The BFs in both non-adapted and adapted tuning are marked as open circles. The colored arrows show the shift directions of the BFs, which were repulsive during adaptation. **(D)** Comparison of the non-adapted and adapted tuning when the adaptors were located on the far flank of the RF (flank adaptor). Using the same conventions as in **(C)**, the BFs shifted attractively toward the adaptors during adaptation. **(E)** The averaged spike waveform (mean ± SD) of the example neuron during a segment of recording. **(F)** FRA of this neuron (bandwidth of 30 dB above threshold, BW_30_ = 0.31 octave, Q_10_ = 6.32, CF = 7.26 kHz). **(G)** The relative changes in the adaptor response (top, ΔR_f = adaptor_), peak response over all frequencies (middle, ΔR_peak_) and shift magnitude of the BF (bottom, Δ_BF_) vary with the adaptor position. A positive Δ_BF_ indicates a repulsive shift while a negative one represents an attractive shift.

A craniotomy was performed to expose the cortex over the right IC contralateral to the side of acoustic stimulation (left side). A supporting bar was attached to the skull to keep the head fixed with the auditory pathway intact. All electrophysiological data were recorded using a Tucker-Davis Technologies system (TDT, Gainesville, FL, USA) in a sound-attenuation chamber. Extracellular spike waveforms were obtained using tungsten electrodes (tip resistance: 2~5 MΩ; A-M systems, Carlsborg, WA, USA; 125 neurons) and silicon probes (1 × 16, spaced at 100 μm, NeuroNexus technologies, Ann Arbor, MI, USA; 136 neurons). Neural signals recorded using both types of electrodes had equal signal quality. Neuronal signals were amplified, filtered (0.3–5 kHz), and digitalized at 25 kHz (TDT). Spike sorting was performed using TDT and Offline Sorter (Plexon, Dallas, TX, USA) online and offline, respectively, to isolate single unit activities (Signal-to-Noise ratio > 14 dB, Figure 1E).

The electrode tracks were labeled by probes coated with the red fluorescent dye DiI (Sigma-Aldrich, St. Louis, MO, USA). Before recording, the probe was immersed in an 80 mg/ml solution of DiI (in 50/50% acetone/methanol) under microscopic control (Magill et al., 2006). After the experiment, the animal was transcardially perfused with saline followed by 4% formalin under deep anesthesia. The brain was removed and placed in 4% formalin for 2 days. Afterwards it was placed in 20% and then in 30% sucrose until it sank. The brain was then blocked and coronal sections (60 μm) were cut on a freezing microtome. Then the recording sites were verified histologically in Nissl-stained brain sections.

### Acoustic stimuli

Acoustic stimuli were generated by the TDT system, sampled at 200 kHz and delivered via an electrostatic speaker (ES1, TDT) 15 cm away from the contralateral ear of the rat along the interaural axis. The speaker was calibrated using a microphone (G.R.A.S Type 40BE, Holte, Denmark) and a measuring amplifier (B&K Type 2610, Nærum, Denmark). All stimuli were presented in free field and both ears of the rat were exposed to the sounds.

A sequence of pure tones (54 ms duration with 4 ms onset and offset ramps) with 51 logarithmically spaced frequencies randomly spreading from 1 to 60 kHz and intensities from 10 to 70 dB SPL with 5 dB steps (Hernández et al., 2005) was presented for measuring the frequency response area (FRA, Figure 1F). The whole sequence was repeated 3 times. For neurons with narrow frequency tuning, we repeated the tone sequence with a smaller frequency spacing. The characteristic frequency (CF) and minimum threshold were visually determined by the frequency and intensity of the lower bound of the FRA.

After characterizing the basic properties of a neuron, three stimulus ensembles were presented: (1) a uniform stimulus ensemble, (2) a biased stimulus ensemble and (3) two oddball sequences. In a uniform stimulus ensemble, the frequency probes were 20 pure tones logarithmically spaced spreading across 1~4 octaves (depending on the width of the FRA of the neuron) centered at the neuron's CF and with the sound level 30 dB SPL above the threshold. The tone of each frequency was repeated 10 times and presented randomly with an inter-stimulus interval (ISI) of 1 s (Figure 1A). There are 200 trials in a uniform stimulus ensemble.

In a biased stimulus ensemble, the same 20 frequency probes as in the uniform ensemble were randomly interspersed among a repeating frequency (adaptor, see Figure 1B). The repeating tones resembled common stimuli in the oddball sequence and accounted for 90% of the entire stimulus sequence. There are 2000 trials in a biased stimulus ensemble. We set the adaptor frequency in two ways: (1) one of the probe frequencies was picked up as adaptor frequency and (2) the frequency for common stimuli in an SSA oddball paradigm was adopted. In the second case, the adaptor was either f_1_ or f_2_ centered at the neuron's BF with BF = (f_1_× f_2_)^1∕2^ (Ulanovsky et al., 2003). The normalized frequency difference (Δf) defined by (f_2_-f_1_)/(f_2_× f_1_)^1∕2^ was 0.1 (0.1442 octave) in this study. The ISI was typically 125 ms (*n* = 261), except for a subset of neurons (*n* = 48), we also tested SSA at a lower repetition rate (ISI = 250, 500 ms) for comparison. The interval between each block was at least 30 s.

A subset of neurons was tested (*n* = 82) using a classic SSA stimulus paradigm, with the oddball sequence consisting of two blocks, each of which contained 400 tones at those two frequencies (f_1_ and f_2_). In block 1, the rare stimuli at frequency f_2_ (10%) were randomly dispersed in the common stimuli at frequency f_1_ (90%). In block 2, the probabilities of occurrence of the two stimuli were inversed such that f_1_ was rare and f_2_ was common.

### Data analysis

Neuronal responses were quantified as the firing rate calculated from spikes in a time window from 0 to 100 ms after the stimulus onset. The frequency-tuning curve was measured as the averaged firing rate at each tone frequency. The frequency range of the RF was defined as the frequency extent that evoked responses higher than 10% of the maximal discharge rate. The upper and lower bounds of this range were determined to be the high-frequency and low-frequency edges, respectively. The spectral distance between edges was quantified as the neuron's bandwidth (BW). To better illustrate the change in responses to frequencies relative to the RF range of the neuron, we measured the width-normalized tuning curve, which was plotted as a function of relative frequency, in which we gave BF the nominal value of zero and the high-frequency and low-frequency edges values of -1 and 1, respectively. For comparison, the adaptor frequency was also transformed to the relative frequency described above (referred to as the adaptor position).

By subtracting the original tuning curve from the adapted one, we obtained the difference signal (DS = Adapted - Original) to show the adaptive change in frequency tuning. The population mean tuning curve was calculated by averaging the width-normalized curves in the neural population. The peak response of each curve was normalized to 1.

The common SSA index (CSI) in the oddball paradigm was defined as (Ulanovsky et al., 2003):

(1) $$\begin{array}{l} \text{CSI}-\text{odd}=\frac{d\left(f_{1}\right)+d\left(f_{2}\right)-s\left(f_{1}\right)-s\left(f_{2}\right)}{d\left(f_{1}\right)+d\left(f_{2}\right)+s\left(f_{1}\right)+s\left(f_{2}\right)}, \end{array}$$

where *d*(*f*_*i*_) and *s*(*f*_*i*_), (*i* = 1, 2) indicate the responses to frequency *f*_*i*_ when it is rare and common, respectively. For comparison, the CSI tested with a biased stimulus ensemble had a similar definition:

(2) $$\begin{array}{l} \text{CSI}-\text{ada}=\frac{p\left(f_{1}\right)+p\left(f_{2}\right)-a\left(f_{1}\right)-a\left(f_{2}\right)}{p\left(f_{1}\right)+p\left(f_{2}\right)+a\left(f_{1}\right)+a\left(f_{2}\right)}, \end{array}$$

where *p*(*f*_*i*_) is the response to frequency *f*_*i*_ when it acts as a probe when adapted by the other frequency and *a*(*f*_*i*_) is response to *f*_*i*_ when it acts as an adaptor. *p*(*f*_*i*_) is compared to *d*(*f*_*i*_) while *a*(*f*_*i*_) is compared to *s*(*f*_*i*_) to explore how this adaptive change of frequency RF correlates with SSA.

### Circuit model

We proposed a two-layer feedforward network model with dynamic connection weights to account for the observed phenomena. The first layer is a set of neural filters (frequency channels) tonotopically arranged according to their center frequencies. The response function of each frequency channel was modeled as a series of standard Gabor functions with different center frequencies as follows (Qiu et al., 2003):

(3) $$\begin{array}{l} G_{i}\left(f\right)=K_{g}\cdot e^{-{\left[2\left(f-f_{i}\right)∕\sigma_{g}\right]}^{2}}\cos\left[2\pi \cdot \Omega_{g}\cdot \left(f-f_{i}\right)\;+\;P_{g}\right],i=1,2,\cdots \;,N, \end{array}$$

where *K*_*g*_, σ_*g*_, Ω_*g*_, and *P*_*g*_ are free parameters and *f*_*i*_ represents the center frequency of the *i*th channel among *N* channels. The parameters *K*_*g*_ and σ_*g*_ correspond to the strength and bandwidth of the frequency profile, respectively; Ω_*g*_ models the distance between the excitatory and inhibitory lobes; *P*_*g*_ is the spectral phase of the frequency profile with reference to the center frequency, providing the alignment of excitation and inhibition relative to the peak of the RF.

The second layer neuron integrates input from each frequency channel, generating the output of the network. It is natural to assume that the profile of integration weights also follows a Gabor function. Thus, the connection strength between the output neuron and *i*th input channel is

(4) $$\begin{array}{l} W\left(f_{i}\right)=K_{w}\cdot e^{-{\left[2\left(f_{i}-f_{0}\right)∕\sigma_{w}\right]}^{2}}\cos\left[2\pi \cdot \Omega_{w}\cdot \left(f_{i}-f_{0}\right)+P_{w}\right], \end{array}$$

Where *K*_*w*_, σ_*w*_, Ω_*w*_, and *P*_*w*_ have the same meanings as in Equation (3); *f*_0_ denotes the center frequency of the neuron. Hence, the original non-adapted tuning can be written as the weighted sum of the G functions with multiple centers in the form of convolution as a function of frequency as follows:

(5) $$\begin{array}{l} R_{NA}\left(f\right)=\frac{K_{0}}{N}\overset{N}{\underset{i=1}{\sum }}W\left(f_{i}\right)\cdot G\left(f-f_{i}\right). \end{array}$$

where *K*_0_ represents the global gain and is normalized by the channel number *N*.

During adaptation, the input channel that is frequently stimulated by the adaptor becomes inhibited, causing a reduction of the output neuron's response:

(6) $$\begin{array}{l} \Delta R\left(f\right)=W\left(f_{r}\right)\cdot G\left(f-f_{r}\right), \end{array}$$

where *f*_*r*_ indicates the adaptor frequency. Therefore, the adapted frequency response is formulated as Equation (5) minus Equation (6):

(7) $$\begin{array}{l} R_{AD}\left(f\right)=\frac{K_{0}}{N}\overset{N}{\underset{i=1}{\sum }}W\left(f_{i}\right)\cdot G\left(f-f_{i}\right)-W\left(f_{r}\right)\cdot G\left(f-f_{r}\right). \end{array}$$

For convenience, the overall suppression strength *K*_*g*_·*K*_*w*_ was modeled as a single parameter *K*. We estimated the optimal parameters (*K*, σ_*g*_, Ω_*g*_, *P*_*g*_, σ_*w*_, Ω_*w*_, *P*_*w*_, and *K*_0_) by fitting Equations (5) and (7) with experimental data using a least square method. Forty frequency channels (*N* = 40) were sampled from the range of [−σ_*w*_, σ_*w*_]. Because the integration weight of each channel was normalized by the channel number ($\frac{K_{0}}{N}$ in Equation 7), the selection of the channel number did not influence the results. The termination tolerance of the least square fitting was set to 10^−6^. The Matlab (the Mathworks, Natick, MA, USA) codes for the model are available at http://dx.doi.org/10.6084/m9.figshare.1536568.

## Results

### The RF change depends on the adaptor position and bandwidth

A total of 261 well-isolated single units were tested with both the uniform and biased stimulus ensembles. Figures 1C,D demonstrate how the preferred frequency and responsiveness of an example cell changed during adaptation to multiple adaptors. The absolute value of the adaptor position was smaller than 1 if the adaptor was inside the RF (center), otherwise it was larger than 1 (flank; see Materials and Methods). When the adaptor position was at a slightly lower frequency than the cell's original BF, the preferred frequency shifted to the higher frequencies (the right side), away from the adaptor (Figure 1C, left). Similarly, when the adaptor position was slightly higher than the original BF, the preferred frequency shifted to the lower frequency (the left side) (Figure 1C, right). This is called a repulsive effect. In both cases, there was a decrease in response at the adaptor frequency as well as in the maximal discharge rate. Interestingly, when the adaptor was far from the original BF (i.e., at the border or outside the RF), the preferred frequency was shifted toward the adaptor. This is called an attractive effect. An adaptor at a left flank position caused the preferred frequency to shift to the left (Figure 1D, left) while the one on the right attracted it to the right (Figure 1D, right). Meanwhile, the peak response and local response at the adaptor frequency were reduced less.

To quantify the relationship between the adaptor position and the magnitude of changes in the tuning curves, the mean value of the following three indicators were measured: the amount of reduction of the response at the adapting frequency (ΔR_f = adaptor_) and of maximal discharge (ΔR_peak_), as well as the magnitude of the shift in the BF (Δ_BF_; Figure 1G). ΔR_f = adaptor_ and ΔR_peak_ decreased in magnitude with the enlargement of the spectral distance between the adaptor frequency and the original BF (Figure 1G, top 2 panels). The BF exhibited a repulsive shift with respect to the adaptor frequency when stimulated with the center adaptor, but remained unchanged or shifted attractively when the spectral distance between the adaptor and original BF was far (Figure 1G, bottom).

**Figure 2:**
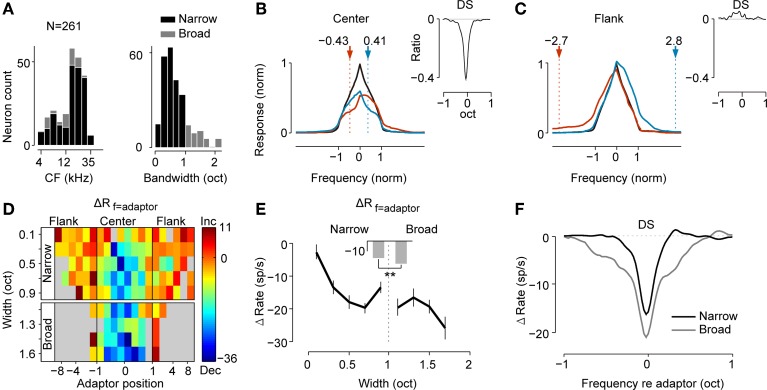
**Population analysis reveals suppression and facilitation in frequency tuning and its dependence on the adaptor position and the bandwidth of the neuron. (A)** Distributions of CF (left) and bandwidth (right) of neurons (*n* = 261). Neurons with BW < 1 octave were defined as narrowly tuned neurons (black, *n* = 214) and others were defined as broadly tuned neurons (gray, *n* = 47). **(B,C)** Averaged original (black) and adapted tuning (colored) when adaptors were located in the center **(B)** or flank **(C)** of the RF. The adaptor was either below (red) or above (blue) the original BF. Each curve was coordinated to the center at the BF and was normalized to its maximal response and bandwidth before averaging. Dashed lines and arrows: the averaged location of adaptors in both cases. Insets: the averaged difference signal (DS) between the original and adapted tuning. DS of each neuron was normalized by the peak firing of the original tuning and was centered at the adaptor before averaging. **(D)** The dependence of the magnitude of local suppression at the adapting frequency (ΔR_f = adaptor_) with the adaptor position (abscissa) and bandwidth of the neuron (ordinate). Narrowly and broadly tuned neurons were separated with 1 octave BW as the boundary. The thick black vertical lines divided the regions with the center and flank adaptor with the RF edge as the boundary. Strong suppression was concentrated in the center region, especially for broader neurons. **(E)** Dependence of the ΔR_f = adaptor_ on the bandwidth of the neuron when the adaptor was within the RF (mean ± SE). The mean values for narrowly and broadly tuned neurons are compared as bars in the middle demonstrating significantly stronger suppression for broadly tuned neurons (Wilcoxon rank sum test, *p* = 2.8 × 10^−6^). **(F)** Mean DSs for narrowly and broadly tuned neurons when adaptors were within the RF (coordinated to the adaptor before averaging). Adaptation caused a significantly broader suppression range for broadly tuned neurons (BW_broad_ = 1.4 octaves, BW_narrow_ = 0.5 octaves, Wilcoxon rank sum test, *p* = 4.5 × 10^−11^).

The distributions of the CF and the BW of all neurons obtained from their FRA are shown in Figure 2A. Neurons with a BW of less than 1 octave were defined as narrowly tuned neurons (*n* = 214), while the others were defined as broadly tuned neurons (*n* = 47) for further comparisons. For the purpose of population analysis, we separated adapted tunings as “center” and “flank” groups according to the relative distance between the adaptor and original BF (Figures 2B,C). The adaptors within the RF range (between nominal frequency -1 and 1) were grouped as “center,” while those outside the range were grouped as “flank.” Each curve was normalized according to its maximal firing rate and BW before averaging (see Materials and Methods for more details).

Consistent with the example neuron previously shown (Figures 1C–G), the adaptor caused frequency-specific suppression or facilitation, which depended on the adaptor position. Center adaptors reduced the maximal response and caused larger suppression near the adaptors (Figure 2B). However, the responses on the farther flank were weakly suppressed or even were facilitated. To better illustrate the change before and during adaptation, we plotted the difference signal (DS) between the adapted and original tuning curve (Figure 2B, inset). The DS revealed that responses near the adapting frequency were heavily suppressed, whereas responses on the farther flank were less suppressed or even enhanced. The half-height width of the suppression pattern was 0.20 octaves and the zero-width was 0.77 octaves. Conversely, adaptation to the frequencies on the far flank led to response facilitation around the adaptor position (Figure 2C).

To understand how the strength of local suppression vary as a function of adaptor position and the BW of the neuron, changes in the adaptor responses (ΔR_f = adaptor_) were averaged and mapped on the plane of these two parameters (Figure 2D). For both narrowly and broadly tuned neurons, stronger suppressions were caused by center adaptors within the RF. In contrast, the facilitations were only brought by flank adaptors. The boundary between suppression and the facilitation region approximated the RF edges (between nominal frequency -1 and 1). This center-suppression and surround-facilitation pattern demonstrated a center-surround dependence on the strength of the local suppression on the adaptor position.

Narrowly and broadly tuned neurons may represent different populations of cells from different nuclei in the inferior colliculus, with narrowly tuned units more likely from the central nucleus and broadly tuned ones from the external or dorsal nuclei (Aitkin et al., 1975; Syka et al., 2000; Duque et al., 2012; Ayala et al., 2013, 2015b). To compare the strength of local suppression between these two groups of neurons, we averaged the ΔR_f = adaptor_ induced by the center adaptors among neurons with similar BWs (Figure 2E). It revealed that broadly tuned neurons underwent stronger local suppression when adapted to the center adaptors (Wilcoxon rank sum test, *p* = 2.8 × 10^−6^). In addition to the strength of local suppression, the frequency range of local suppression between narrowly and broadly tuned neurons was also compared. We plotted the mean DS for these two types of neurons and demonstrated that both the amount of the decrement and frequency extent of local suppression were larger for broadly tuned neurons (Figure 2F). The mean DS of broadly tuned neurons had a significantly larger bandwidth (Wilcoxon rank sum test, *p* = 4.5 × 10^−11^) than the narrowly tuned ones. This difference suggested that broadly tuned neurons may undergo stronger and more widespread adaptation than narrowly tuned ones do.

### Adaptation magnitude displays a center-surround profile

From the example neurons (Figure 1) and the population analysis (Figure 2), we found that the adapted tuning exhibits inhomogeneous change, including local suppression or facilitation, change in the peak response, and a shift of the BF. To further characterize the profile of this change, we quantified the following changes with respect to the adaptor position: change in the ratio of the adaptor response (ΔR_f = adaptor_), change in the ratio of the peak response (ΔR_peak_) and the shift in magnitude of the BF (Δ_BF_).

**Figure 3:**
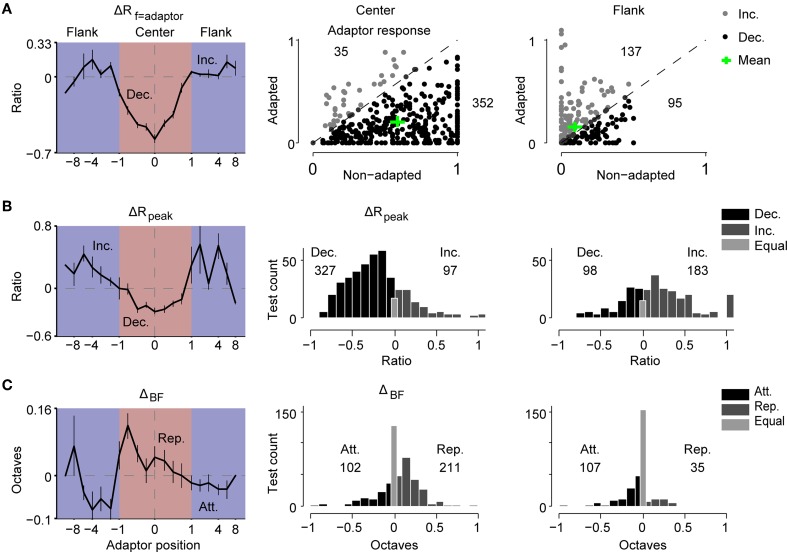
**The magnitude of the adaptive change of the RF displayed a center-surround pattern. (A)** Left: the profile of the change ratio of the responses at the adaptor (ΔR_f = adaptor_) with respect to the adaptor position. ΔR_f = adaptor_ was normalized by the individual peak response of the non-adapted tuning. Middle and right: response at the adaptor frequency in the adapted condition against the original condition for each test (normalized by the individual peak response of original tuning) when the adaptor was in the center (middle panel) or on the flank of the RF (right panel). The mean value is indicated by a green cross. The number of tests showing increasing (gray) or decreasing (black) responses is annotated above or below the diagonal, respectively. **(B)** Left: the profile of the change ratio of the maximal response (ΔR_peak_) with respect to the adaptor position. ΔR_peak_ was normalized by the individual maximal response of original tuning. Middle and right: the distributions of ΔR_peak_ when adaptors were in the center (middle panel) or on the flank (right panel). The numbers denote the number of tests with decreased (Dec.) and increased (Inc.) responses. **(C)** Left: the profile of the shift magnitude of the BF (Δ_BF_) with respect to the adaptor position. Positive values indicate repulsive shifts (Rep.) while negative values represent attractive shifts (Att.). Middle and right: the distributions of Δ_BF_ when the adaptors were in the center (middle panel) or on flank of the RF (right panel). The numbers denote the number of tests with attractive and repulsive shifts. All error bars indicate the mean ± SE.

First, we investigated the relationship between the change in the adaptor response (ΔR_f = adaptor_) and the adaptor position (Figure 3A). The suppression of adaptor responses becomes gradually released when the adaptor moves away from the RF center, and turns into a slight increment when the adaptors are outside the RF (Figure 3A, left). To explore the distributions of response changes at the adapting frequency in the neural population, the adaptor response in each adapted tuning was plotted against that in the corresponding non-adapted tuning for the case of center adaptors (Figure 3A, middle) and flank adaptors (Figure 3A, right). Decreases were observed in the majority of the tests with center adaptors (91%, 352/387) and in the minority of tests with flank adaptors (41%, 95/232). The mean percentages of adaptor response change measured with biased ensemble relative to that measured with uniform ensemble were −34% and 175%, respectively (see the green crosses in Figure 3A right two panels).

Meanwhile, we observed similar center decrements and surround increments with regard to the adaptor position for peak responses changes during adaptation (Figure 3B, left). The distribution of the change ratio of the peak response (ΔR_peak_) in the case of center adaptors (Figure 3B, middle) was compared to that of the flank adaptors (Figure 3B, right) in the neural population. It displayed a bias toward decrements of peak responses for the center adaptors (Figure 3B, middle) and a bias toward increments for flank adaptors (Figure 3B, right).

Furthermore, the dependence of the shift magnitude of the BF (Δ_BF_) on the adaptor position also presented a center-surround pattern in that the center adaptors repelled the BF away from them, while the flank adaptors attracted the BF toward them (Figure 3C, left). These were also supported by the distribution of Δ_BF_ in the neural population, showing a more repulsive shift of the BF for center adaptors (Figure 3C, middle) and a more attractive shift for flank adaptors (Figure 3C, right).

In addition, the center adaptors sharpen the tuning curves, while the flank adaptors slightly widen the tuning curves (Supplementary Figure 1). These phenomena imply that the change magnitude caused by adaptation is weighted depending on the spectral distance between the adaptor frequency and original BF. The center adaptors elicit a bigger size of change effects, while the flank adaptors evoke smaller or opposite effects, displaying a center-surround profile.

### A two-layer feedforward model explains the frequency-specific adaptation

**Figure 4:**
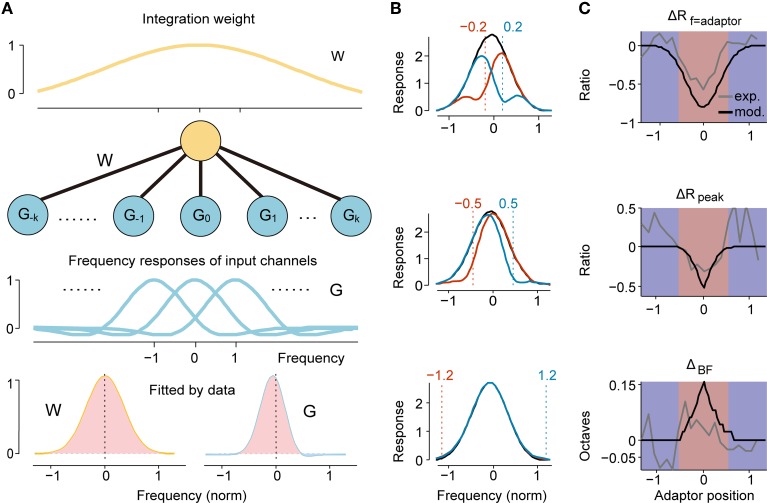
**Two-layer feedforward network model predicted RF Change. (A)** The architecture of our two-layer feedforward network. The upper neuron integrates weighted inputs (W function, yellow line in top panel). Each channel in the lower input layer has a specific frequency response organized tonotopically (G function, blue lines). When a specific frequency is repeatedly presented, the input from the adaptor channel is suppressed, resulting in a change in the output as observed in the upper layer neurons. Both W and G are modeled as Gabor functions with individual parameters. Bottom: the W function and G function with parameters fitted by experimental data of narrowly tuned neurons. Light red and light blue indicate excitatory and inhibitory sub-regions, respectively. **(B)** The simulated adapted (colored) and original tunings (black) by six different adaptors qualitatively depict the experimental observation. The normalized frequency of each adaptor is annotated with a dashed line and the text above it. **(C)** The profile of the ratio of change in adaptor response (top), peak response (middle) and shift magnitude (bottom) with respect to the adaptor position from model simulation (black) and the experiment (gray) match well in the overall center-surround pattern.

In fact, from the above analysis, we can find two levels of inhomogeneous patterns: one is centered at the adaptor frequency (shaped as the DS signal shown in Figures 2B,F) and the other is centered at the BF of the original tuning (shaped as a center-surround profile in Figure 3). It is tempting to fit these two patterns with appropriate radial functions and to expect the observed RF change to be explained by the convolution of these two levels of function. Here, we proposed a two-layer feedforward network model as a plausible neural circuit that gives rise to the dynamic change in frequency tuning of IC neurons (Figure 4A). This model contains a layer of input channels, each of which has a frequency tuning profile (referred to as the G function) with a specific center frequency organized tonotopically, and it connects to the output neuron with different weights (referred to as the W function). The center-suppression and surround-facilitation structure with regard to the adaptor in DS was described as the G function, the frequency profile of an adaptor channel. Meanwhile, the W function should be largest in the center and smaller or negative in the surround to depict the strength of the adaptation effect for each channel (Figure 3, left column). A Gabor function can capture these characteristics well; hence, both G and W functions were modeled as Gabor functions (Qiu et al., 2003) but with different parameters as described in the Materials and Methods section. The suppression strength *K* = *K*_*w*_ ·*K*_*g*_, the bandwidth σ_*g*_, σ_*w*_, the spectral density Ω_*g*_, Ω_*w*_ and the spectral phase *P*_*g*_, *P*_*w*_ for the G or W function and the global response gain *K*_0_ are free parameters and were fitted with both original and adapted frequency response data in each trial for each neuron using a least-square method. Considering frequency-specific adaptation induced different strengths and bandwidths of local suppression in narrowly and broadly tuned neurons (Figures 2E,F), we fitted the model with data from these two groups separately.

Because narrowly tuned neurons accounted for a majority of the data (214/261) and were tested more comprehensively with adaptors both inside and outside the RFs (Figure 2D), we first fitted the model to narrowly tuned neuron data. The fitted parameters of W and G function were summarized in Table 1. The fitted G function was asymmetric such that it had a larger excitatory response area expanding in the lower frequency side and a sharper excitatory area with an inhibitory upper sideband (Figure 4A, bottom right). It captured the characteristics of the DS well (Figure 2F, black). The W function proximately degraded into a Gaussian function due to strong attenuation in the lateral inhibition lobe (Figure 4A, bottom left). It had much lower spectral density (Ω_*w*_ < Ω_*g*_) and a slightly broader bandwidth (σ_*w*_ > σ_*g*_), which suggests that the neuron integrates multiple input channels (G function). Simulations of the original and adapted tuning curves (Equations 5 and 7) with six adaptors (normalized frequency: ± 0.2, ± 0.5, and ± 1.2) predicted the local suppression induced by adaptation and the dependence of the change magnitude on the adaptor position (Figure 4B). The facilitation and attractive shift evoked by the flank adaptors were not obvious in simulations (Figure 4B, bottom), which may be explained by the lack of a significant inhibitory sideband in the fitted W function. The magnitudes of changes in adaptor responses (ΔR_f = adaptor_), peak discharge rates (ΔR_peak_) and shifts of BF (Δ_BF_) again exhibited a center-surround organization with respect to the adaptor position, which resembled the experiments well (Figure 4C).

**Table 1:** **The optimal parameters of W and G function fitted with narrow and broad group of neurons**.

	***K***	**σ_*g*_**	**Ω_*g*_**	***P*_*g*_**	**σ_*w*_**	**Ω_*w*_**	***P*_*w*_**	***K*_0_**
Narrow	27.6	0.31	0.64	0.64	0.41	0.33	−0.11	52.1
Broad	62.5	0.69	0.02	1.20	1.30	0.07	0.37	92.0

We also fitted the model with responses of broadly tuned neurons for comparison. The strength of the suppression fitted with broadly tuned neurons (*K* = 62.5) was stronger than that fitted with narrowly tuned neurons (*K* = 27.6), which is compatible with the experimental data (Figures 2E,F). Meanwhile, the fitted bandwidths of the G and W functions and their ratio (σ_*w*_∕σ_*g*_ = 1.9, Table 1) were all larger than those of the narrowly tuned neurons (σ_*w*_∕σ_*g*_ = 1.4, Table 1), suggesting that broadly tuned neurons may integrate more frequency channels and have a broader adaptable frequency range.

### Adapted frequency tuning predicts SSA

When exposed to an oddball stimulus sequence with unbalanced probability of two tones, the neurons in the IC show SSA, in which rare stimuli elicit stronger responses than common ones (Malmierca et al., 2009; Zhao et al., 2011; Duque et al., 2012; Pérez-González and Malmierca, 2012; Pérez-González et al., 2012; Anderson and Malmierca, 2013; Ayala and Malmierca, 2013; Ayala et al., 2013). The common stimulus in the SSA oddball sequence had the same presentation probability as the adaptor in our biased stimulus ensemble. Thus, it had a decreased response due to adaptation, whereas the rare stimulus in the oddball sequence resembled a probe away from the adaptor. Therefore, it evoked a less suppressed or a facilitated response. These features led to larger responses to rare stimuli than to common stimuli. Thus, from the observed tuning changes after adaptation, we can predict the size of the SSA at these frequency combinations.

**Figure 5:**
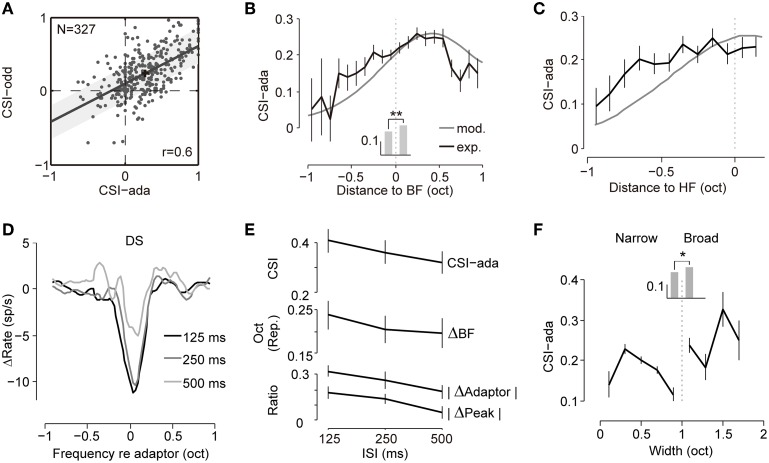
**The adaptive frequency response predicts the SSA. (A)** Scatter plot showing CSIs measured with a biased ensemble (CSI-ada) and oddball sequence (CSI-odd) were significantly correlated (Pearson's *r* = 0.602, *p* = 1.25 × 10^−33^). The best-fit linear regression line is shown (least square, slope = 0.52). The shadow bounds the 95% confidence interval. The black cross represents the mean value of the dot clusters. **(B)** Averaged CSIs (CSI-ada) calculated from both adapted tunings (black) and the model (gray) as functions of the center of the tested frequency pair (relative to the BF). The curve from the model (maximal value: 0.61) was rescaled to match the maximal value of the curve from the experiment (0.26) for display only. Mean CSIs at frequencies below and above the BF are displayed as bars in the middle. The asterisks indicate that CSIs in the high-frequency group were significantly larger than in the low-frequency group (Wilcoxon rank sum test, *p* = 1.5 × 10^−4^). **(C)** CSIs were grouped according to the frequencies relative to the high-frequency (HF) edge of each cell. Higher CSI values are clearly skewed toward the higher edge. The same conventions as in **(B)**. **(D)** Averaged DSs under three ISIs (125, 250, and 500 ms, coordinated to the adaptor before averaging, *n* = 48) showing greater local suppression under a shorter ISI. **(E)** The dependence of CSI-ada (top), BF repulsive shift magnitude (Δ_BF_, middle), suppression magnitude of the peak (|ΔR_peak_|, bottom) and adaptor response (|ΔR_f = adaptor_|, bottom) on the ISI. It displayed a trend of decreasing effect with increases in the ISI. Spearman's correlation between ISIs and the parameters are -0.08, -0.04, -0.13, -0.15 for CSI-ada, Δ_BF_, |ΔR_peak_|and |ΔR_f = adaptor_|respectively. **(F)** Dependence of CSI-ada on the bandwidth. The mean values for narrowly and broadly tuned neurons are compared as bars in the middle. Asterisk shows that broadly tuned neurons exhibited higher SSA degrees (Wilcoxon rank sum test, *p* = 0.038). All error bars indicate the mean ± SE.

Therefore, we measured the strength of the SSA (common SSA index, CSI) from both the adapted tuning (CSI-ada, 90%/0.5%, ISI = 125 ms) and the oddball paradigm (CSI-odd, 90%/10%, ISI = 125 ms) as defined in the Materials and Methods (Figure 5A). Linear regression of these two measurements exhibited a strong correlation (Pearson's *r* = 0.60, *p* = 1.25 × 10^−33^). The CSI-ada was larger than the CSI-odd, which may result from much lower probabilities for rare stimuli (0.5%) in our biased stimulus set than that in the typical SSA stimulus set or more repetition of adaptor in biased ensemble (1800 trials) than that in oddball sequence (360 trials). To explore whether this increase is due to increase of response to probe tone or decrease of response to repetitive tone, we compare the strength of responses between probe and rare tones, and between adaptors and common tones (Supplementary Figure 2). We found that the probe responses and rare responses were comparable. But the responses to repetitive tones in biased ensemble tend to be weaker than common sound responses in oddball paradigm. The difference may be caused by more repetition of adaptor in the biased ensemble. In general, these results suggested that the SSA degree can be predicted well by the adapted tuning curve.

Additionally, the SSA strength in the IC was previously shown to be dependent on the position of the frequency pair. A high SSA was biased toward the high-frequency and low-intensity regions of the RF (Duque et al., 2012). Here, the inhomogeneous RF change elicited by the adaptor at different positions (Figure 3) may imply an inhomogeneous frequency-specific adaptation across frequency-receptive fields. To test this hypothesis, we chose the adaptor and one of the probes as the common and rare stimulus pair in the SSA oddball sequence to calculate the SSA strength in our adaptation paradigm (CSI-ada). To explore the dependence of the SSA strength on the center frequency of the stimulus pair, we grouped the center frequency (relative to the BF) of each frequency pair (0.1 < |*f*_2_ − *f*_1_| ≤ 0.2 octave) into 0.1-octave-wide bins and averaged the CSI-ada of frequency pairs for each bin (Figure 5B). The SSA strength increased with the center frequency but saturated at approximately 0.6 octaves above the BF (Figure 5B, black line). The simulation of our two-layer network model predicted a similar trend (Figure 5B, gray line). The CSI values for frequencies above the BF were significantly larger than those for frequencies below the BF (Wilcoxon rank sum test, *p* = 1.5 × 10^−4^, Figure 5B, gray bars). If high-frequency pairs, in spite of their relative position within receptive field, elicit stronger SSA degree, we would expect a similar asymmetry as shown in Figure 5B. To investigate the relationship of SSA degree with relative frequency within receptive field and to rule out the bias due to high-frequency pairs, we plotted the CSI against the distances of the center frequency to the high-frequency edge of the tuning (see Materials and Methods) using both experimental data and model simulation. The same trend held true that the CSI increased with closer distances to the higher edge and saturated around it (Figure 5C). Thus, both our observed data and proposed model predicted the bias of the SSA strength toward high frequencies, which was in line with a previous study (Duque et al., 2012). Additionally, it is easy to observe that the asymmetry of the G function in our network model (Figure 4A) leads to the asymmetry of the SSA strength. This may hint at the causes of the frequency asymmetry of the SSA.

If the observed changes, e.g., local suppression, shift of BF and reduction in peak responses, are truly elicited by adaptation, how quickly a neuron becomes adapted may influence the effect. The inter-stimulus interval (ISI) is an indicator that can be used to quantify how rapidly a neuron becomes adapted in that a shorter ISI corresponds to a faster adaptation rate. To evaluate the influence of the adaptation rate, difference signals (DSs) caused by the adaptor at the same position under different ISIs (125, 250, and 500 ms, *n* = 48) were averaged and compared. It is clear that shorter ISIs or faster repetition rates cause larger adaptation strength and a broader frequency range of local suppression (Figure 5D). To compare the change in magnitude under different ISIs, we again quantified the three parameters, namely the amount of response reduction at the adapting frequency (|ΔR_f = adaptor_|), the amount of reduction of the peak firing rate (|ΔR_peak_|), and the magnitude of the repulsive shift in the BF (Δ_BF_) and compared across different ISIs. All three parameters decreased monotonically with an increase in the ISI (Figure 5E). The strength of suppression in the model (*K* in Equation 6) fitted with the same neurons was larger for shorter ISIs (*K* = 62, 58, and 57 for ISI = 125, 250, and 500 ms, respectively), suggesting again that greater adaptation can be induced by faster adaptation. To further clarify the relationship of the frequency-specific adaptation strength and adaptation rate, we compared the CSI-ada under different ISIs and found that the index increased with faster repetition rates (Figure 5E). In general, adaptation under shorter ISIs (higher repetition rates) elicited a stronger adaptation effect, which agreed with the findings in SSA studies that the response discrepancy between rare and common stimuli were larger for shorter ISIs (Ulanovsky et al., 2003; Antunes et al., 2010; Zhao et al., 2011).

Moreover, as previously stated, adaptation caused stronger and wider local suppression in broadly tuned neurons (Figures 2E,F). This result implies that broadly tuned neurons exhibit higher SSA degrees. Here, we compared the CSI values (CSI-ada) of neuron groups with different bandwidths, and confirmed that broadly tuned neurons exhibited stronger adaptation compared to narrowly tuned neurons (Figure 5F, Wilcoxon rank sum test, *p* = 0.038), which agreed with previous SSA studies (Malmierca et al., 2009; Duque et al., 2012; Ayala et al., 2013, 2015b; Ayala and Malmierca, 2013).

## Discussion

In this study, we compared the frequency-tuning curve measured with a uniform stimulus ensemble and biased ensemble in the auditory midbrain and found that adaptation to frequencies within the RF caused local suppression of the frequencies near the adaptor tone and a repulsive shift of the BF. The dependence of the magnitude of the change on the adaptor position exhibited a center-surround organization in that the center adaptors elicited stronger effects, while the flank adaptors induced a small or opposite effect. Furthermore, we proposed a two-layer feedforward model that could qualitatively account for the observed phenomena with parameters fitted to the experimental data. Importantly, the adapted frequency tuning in both the experiments and model were able to well predict IC responses to classic oddball sequences. These results revealed the characteristics of the dynamic frequency-receptive field induced by frequency-specific adaptation.

This study also introduced a unique approach toward neural network perturbation. Among a large sample of hundreds of neurons with diversified tuning frequencies and bandwidths, their receptive fields were probed by biased stimulus ensemble with sets of frequency adaptors. The dynamic changes in their frequency tunings were systematically examined and captured by a two-layer converging network. This combination of massive neuronal perturbation and network modeling provided insights into neural network connections and plausible circuits in the auditory midbrain.

### Dynamic changes of frequency responses in the auditory system

In the auditory system, frequency response plasticity in the auditory cortex can also be induced by classical conditioning (Condon and Weinberger, 1991; Malone and Semple, 2001) or attentional tasks (Fritz et al., 2003). Moreover, shifts of the BF in the IC can be elicited by electrical-stimulation in auditory cortical neurons via the corticofugal pathway (Suga and Ma, 2003). The direction of the shift could be either centrifugal (repulsive) or centripetal (attractive) depending on the distance between the center frequencies of stimulated neurons and observed neurons (Suga and Ma, 2003; Xiao and Suga, 2005). These studies were concerned only with top-down modulation of frequency tuning. For the bottom-up direction, context-dependent facilitation of a specific frequency other than the CF was also observed in the spectrotemporal receptive field evoked by a narrowband sound in the auditory cortex (Gourévitch et al., 2009). A previous study provided the first evidence of changes in frequency tuning induced by adaptation in the auditory cortex (Ulanovsky et al., 2003). A recent study used similar protocol and found adaptation unevenly suppressed the tuning curves and shifted the tuning curves with an adaptor-dependent manner in auditory cortex (Parto Dezfouli and Daliri, 2015). In the current study, we systematically investigated the adaptive change in frequency tuning caused by an unbalanced stimulus sequence in the auditory midbrain for the first time.

The context effect of sound history has been extensively studied by assessing neural sensitivity to sequences of two tones, which is known as forward masking. The changes in frequency tuning reported here share some similarities with masked frequency responses. For instance, both suppression and facilitation depending on the contrast between the masker (preceding sound) and probe (succeeding sound) were observed in the auditory cortex (Bartlett and Wang, 2005; Scholes et al., 2011) and IC (Finlayson, 1999; Malone and Semple, 2001). Masker-induced CF shifts in the FRA were also found in the auditory cortex (Peng et al., 2010). Greater modulation was found when the masker was closer to the CF or the ISI was shorter (Peng et al., 2010), which was in line with our findings. These results suggest that frequency-specific adaptation and forward masking may partly share common mechanisms based on the dynamics and center-surround arrangement of neural connections.

### Neural circuitry for frequency processing in the auditory midbrain

Optogenetic perturbation has been widely used as a tool to reveal the functional connections between various neural circuits (e.g., Adesnik et al., 2012; Olsen et al., 2012; Wilson et al., 2012; Sturgill and Isaacson, 2015). Optogenetics are very effective to dissect cell types with different genetic markers and are powerful for fast manipulation of a group of cells. Here, we proposed another manipulation approach—adaptation—to infer connectivity within the subcortical auditory processing circuits in which neurons may not be differentiated according to the genetic content. The adaptation method systematically manipulates the functional connectivity in a restricted part and when put together, it helps to form a picture of the underlying structure. Thus, we are trying to perturb the system using stimulus adaptors and probes and to fit the recorded data into a network model with the hope of revealing a plausible circuit structure for the ultimate dissection of the detailed connections. The feedforward network we proposed here has been widely hypothesized to form the basis of neuronal receptive fields since early classical visual research (Hubel and Wiesel, 1962). A similar integrative model proposed to predict contextual modulation of sensory responses (Lochmann et al., 2012) further supports the idea that the RF can be shaped by neural integration and can unveil the local circuits. The ability to reshape the RF dynamically makes the adaptation approach a useful tool for probing the underlying circuit for sensory processing. Our model suggests that spectral RF is a malleable profile characterizing the IC neural circuit and reflecting the frequency response properties of the afferents to the IC, including the tonotopic structure, bandwidth of the frequency channel and organization of the excitation and inhibition.

**Figure 6:**
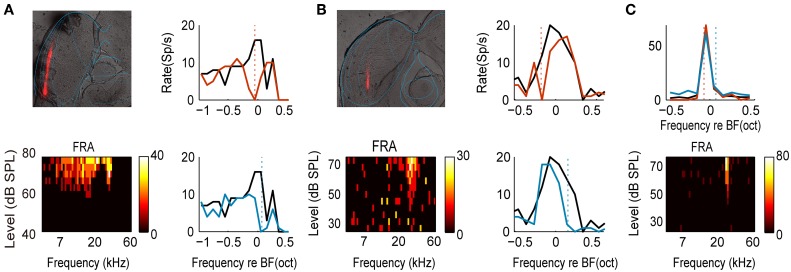
**The examples of two recording sites and a non-adapted neuron. (A)** An example of recording site in external cortex of IC. Top left: brain section (60 um). Red track: DiI red fluorescent mark of electrode track. Blue: rat brain atlas outline (adapted from Paxinos and Watson, 2007). Bottom left: FRA of one example neuron from this site (CF = 17.3 kHz, BW_25_ = 2.66 octaves). Right: frequency tuning curves measured with uniform (black) and biased ensemble (colored) with two adaptors. The dashed vertical lines indicate the adaptor positions. **(B)** An example of recording site in central nucleus of IC. Same convention as in **(A)**. CF = 28.1 kHz, BW_30_ = 0.70 octave. **(C)** An example of non-adaptive neuron. Top: frequency tuning curves measured with uniform (black) and biased ensemble (colored) with two adaptors. The dashed vertical lines indicate the adaptor positions for corresponding colored curves. Bottom: FRA of this neuron (CF = 26.6 kHz, BW_30_ = 0.16 octaves).

IC can be segregated into central nucleus (CNIC) and cortical regions (Loftus et al., 2008), which are considered to be involved in lemniscal and non-lemniscal pathway, respectively (Rouiller, 1997). Our results (Figure 5F) and previous studies suggested a weaker adaptation in neurons in CNIC which have narrow bandwidths (Duque et al., 2012; Ayala et al., 2013, 2015b). Adaptation is even absent for some neurons in CNIC (e.g., Figure 6C). However, we found adaptive neurons in both central nucleus and cortical regions (Figures 6A,B). Neurons within the lemniscal subdivision mainly receive inputs from brainstem which preserve topographic relations in the various afferent populations (Winer and Schreiner, 2005; Loftus et al., 2010). For these neurons, the first layer in the model resembles this brainstem inputs which can be specifically suppressed at the level of CNIC. The tonotopic organization of the input channels is supported by the existence of frequency-band lamina in CNIC (Stiebler and Ehret, 1985). Intriguingly, the bandwidth of the input channel (σ_*g*_ = 0.31 in the model) matches the bandwidth that a single lamina covers (~0.3 octaves in rat, Malmierca et al., 2008) and might provide the substrate for the emergence of critical bands that limit the perceptual frequency resolution (Ehret and Merzenich, 1985; Schreiner and Langner, 1997). The adaptation in lemniscal pathway, though weak, can be accounted for by specific suppression of brainstem inputs.

Although isofrequency laminae outside the CNIC seem to be absent, the neurons in dorsal cortex do form layers (Aitkin, 1986). And recent evidence showed that tonotopic arrangement also exist in dorsal IC neurons (Barnstedt et al., 2015). Neurons in cortical IC were demonstrated to have broader tuning and receive inputs from both CNIC and cortical feedback (Aitkin and Phillips, 1984; Winer et al., 1998, 2002; Winer and Schreiner, 2005; Stebbings et al., 2014; Ayala et al., 2015b). They may integrate several CNIC inputs which are tonotopically arranged. In this case, the first layer of our model may correspond to projection from the CNIC. Since the adaptation at this level is the second level upon the basis of the CNIC, it is reasonable that the adaptation strength is stronger than that of CNIC. In addition, they also receive feedback from cortex (Winer et al., 1998; Ayala et al., 2015b), which may enhance and modulate the SSA in non-lemniscal pathway (Anderson and Malmierca, 2013; Malmierca et al., 2015). A recent study show that the SSA neurons in the non-lemniscal subdivisions receive strong inputs from auditory cortical areas (Ayala et al., 2015b). The contribution of corticofugal projection cannot be accounted for by current feedforward model.

The two-layer center-surround arrangement of the frequency-specific suppression and facilitation were incorporated in the shape of the G (the first layer) and W functions (the second layer) in the model. In one of the previous studies, Gaussian functions were used as gain profiles to account for similar stimulus-specific and neuron-specific effects in adaptive changes of orientation tuning in the visual cortex (Benucci et al., 2013). Here, we used Gabor functions (Qiu et al., 2003), which can degrade into Gaussian functions and have additional advantages in depicting the center-surround arrangement (Figure 4A). The fact that cochlear nucleus neurons did not show frequency-specific adaptation (Ayala et al., 2013) suggests that the frequency-specific suppression reported here is not inherited from lower nuclei. Furthermore, it cannot be accounted for by spike habituation or neural fatigue alone given that the flank adaptor, which did not elicit spikes by itself, still impacted tuning (Calford and Semple, 1995; Brosch and Schreiner, 1997; Bartlett and Wang, 2005). And it also cannot account for bidirectional shifts in BF caused by center adaptors. The tuning changes reported here could be recovered within 30 s (Figure 1C, gray curve) and, therefore, were not caused by long-term depression or long-term potentiation that may last for tens of minutes or more (Kessels and Malinow, 2009; Friauf et al., 2015). Instead, it may result from the enhancement of inhibitory inputs or synaptic depression of excitatory inputs at the level of the IC. If inhibition enhancement is involved, the inhibition should be frequency-tuned. GABAergic inputs were previously demonstrated to primarily affect within RFs (Palombi and Caspary, 1996) of target IC neurons but they also affects sidebands (Wang et al., 2000; Metherate, 2011) and may be involved in shaping the frequency-specific suppression or facilitation reported here. For instance, GABAergic inputs from the dorsal nucleus of the lateral lemniscus are tonotopically aligned with IC target neurons and may be one possible source of inhibition for neurons in CNIC (Merchán et al., 1994). While for neurons in cortical IC, the inhibition may be driven by direct inputs from local inhibitory neurons and/or through di-synaptic connections of auditory cortex neuron (Winer et al., 1998; Stebbings et al., 2014). The existence of abundant inhibitory neurons and interconnections in the intrinsic circuit within the CNIC may provide an intrinsic source of inhibitory input (Saldaña and Merchán, 1992). For cortical projection, since corticofugal projections are known to be glutamatergic (Feliciano and Potashner, 1995), di-synaptic connections that glutamatergic neurons contact local inhibitory neurons may be involved. The synaptic depression of excitatory inputs is also a possible mechanism. A recent study shows that the blockade of glutamate receptors mediates an overall decrease of the neural response which support the gain control effect elicited by excitatory inputs (Ayala et al., 2015a). Note that the depression of excitatory inputs alone cannot account for the facilitation at lateral frequencies (Figure 2C). The frequency profile with lateral inhibition of the adapted input channel in the model (G function) suggested that depression of lateral inhibitory inputs may be involved, and post-inhibitory rebound or disinhibition with considerable subthreshold processing of neuronal inputs may underlie this depression (Dragoi et al., 2000; Xie et al., 2007). The dorsal cochlear nucleus type III cell has a frequency-receptive field with lateral inhibition and may be one candidate as input (Young and Calhoun, 2005). Another possible mechanism to account for the facilitation elicited by flank adaptors may be that repetitive presentation of flank adaptors accumulatively depolarized the neuron's membrane potential until reaching the spike threshold (Metherate, 2011). In general, these results may provide insights into the response properties of neural inputs to the IC and intra-collicular connections in the IC.

### Functional implications

In the present study, the responses to the biased ensemble exhibited suppression with high-probability stimuli and facilitation with low-probability stimuli. The discharge energy was redistributed and tended to be equalized across different statistical content of the stimuli. This helps to maximize the coding efficiency of a sensory system with a fixed dynamic range (Dean et al., 2005; Sharpee et al., 2006; Wark et al., 2007; Zhao and Zhaoping, 2011; Benucci et al., 2013). Similar adaptive tuning changes in the visual system results in enhancement of the capacity to discriminate local features (Müller et al., 1999; Dragoi et al., 2002; Krekelberg et al., 2006). And in human auditory cortex, increase in the frequency specificity induced by an adaptor stimulus was observed (Briley and Krumbholz, 2013). Our results also suggest that the tuning curves are sharpened by center adaptors during adaptation (Supplementary Figure 1). The frequency-specific suppression/facilitation pattern enhances the local contrast between the adaptor and surrounding frequencies by increasing the slope between them, potentially improving the neuron's capacity for discriminating neighboring frequencies and detecting novel sounds.

The RF changes reported here can predict the SSA well, and, therefore, hint at possible underlying mechanisms. The SSA has not been found in the cochlear nucleus and might first emerge in the IC (Ayala et al., 2013). It is not generated by the corticocollicular projection through top-down modulation (Anderson and Malmierca, 2013; Malmierca et al., 2015). The predictive power of our model suggests that frequency-specific adaptation may be generated via a feedforward network, employing synaptic depression (Duque et al., 2015) of the afferents either from CNIC to cortical IC neurons or from a lower nucleus to CNIC. It is a variant of the adaptation of narrowly tuned modules (ANTM) model (Nelken, 2014), which was proposed previously (Mill et al., 2011a,b, 2012; Taaseh et al., 2011; Hershenhoren et al., 2014). In cellular level, the frequency-specific integration may be generated by dendritic processes. Evidence for such a mechanism was recently described in an insect auditory interneuron that afferents, tuned to different frequencies, connect with different parts of the neuron's dendrite (Prešern et al., 2015). In addition to the convergence of depressing synapses that convey frequency-specific inputs, our model also incorporates surrounding inhibition. Broadly tuned neurons exhibited more extensive suppression and larger SSA strength (Figure 5), which is consistent with the stronger SSA found in the non-lemniscal pathway in which neurons have broader tunings (Malmierca et al., 2009; Duque et al., 2012, 2015; Ayala and Malmierca, 2013; Ayala et al., 2015b). This result implies that broadly tuned neurons might undergo more prominent adaptation due to broader convergence of inputs, including those from the CNIC or cortical feedback that cannot be fully covered by the present model (Ayala et al., 2015b). Though the ANTM model, similar to ours, predicts the responses in the IC well, it failed to predict some features of the cortical SSA (Taaseh et al., 2011; Yaron et al., 2012; Nelken et al., 2013; Hershenhoren et al., 2014). For instance, it failed to predict that responses to rare tones embedded in sequence of common tones were stronger than when they were presented alone (Taaseh et al., 2011; Hershenhoren et al., 2014). It also failed to predict the fact that responses to rare tones in random sequence were higher than those in periodic sequence with the same probability (Yaron et al., 2012). Additionally, it cannot account for the SSA tested with complex spectral-temporal patterns reported in cortex (Nelken et al., 2013). These features are suggested to be absent in IC (Khouri et al., 2013). A different network structure should be considered for these cortical features, which are regarded as “true deviances” (Nelken, 2014).

Our results suggested that disinhibition can further enhance the SSA effect via facilitation of distant rare frequencies. The enhancement of deviant responses was also observed in *ex vivo* networks of cortical neurons and was abolished by blocking GABAergic inhibitory transmission (Eytan et al., 2003). In the IC, GABA_A_-mediated inhibition was shown to enhance the SSA through a gain control mechanism and further refine and sharpen the SSA (Pérez-González and Malmierca, 2012; Pérez-González et al., 2012). And glutamaterigc inputs were also shown to affect SSA through gain control mechanism (Ayala et al., 2015a). The role of neural modulation factors including GABA_A_-mediated inhibition on facilitation at remote rare frequencies needs to be further investigated.

The asymmetry of the low- and high-frequency lobes in a fitted G function (Figure 4A) contributes to the asymmetric SSA strength across frequencies in the model and may underlie the similar phenomenon observed in current (Figures 5B,C) and previous studies (Duque et al., 2012). Note that the typical tuning curve of auditory nerve fibers is strongly asymmetric, with steep high-frequency slopes and shallow low-frequency slopes (Ehret and Schreiner, 2005) resembling the fitted G function. And this kind of low-tilted tuning RF is also very common in the CNIC (Ehret and Schreiner, 2005), which may provide a possible afferents to the neurons in non-lemniscal subdivisions. These characteristics of input to the IC may contribute to the inhomogeneity of SSA across frequencies.

In conclusion, by perturbing the auditory midbrain neurons with frequency adaptors, inhomogeneous changes in frequency tuning were observed and modeled as a feed-forward network, which may contribute to the function of novelty detection and shed light on the underlying circuits and networks.

## Author contributions

The study was conceived and designed by BH. LS and LZ did the experiments. LS analyzed the data. LS, LZ, and BH wrote the manuscript.

### Conflict of interest statement

The authors declare that the research was conducted in the absence of any commercial or financial relationships that could be construed as a potential conflict of interest.
