# Supplementary material for: Frequency-specific adaptation and its underlying circuit model in the auditory midbrain
Source: Front Neural Circuits. 2015 Oct 1;9:55. doi: 10.3389/fncir.2015.00055 (PMC4589587; doi:10.3389/fncir.2015.00055)
Supplement: Supplementary file 1 [file Image1.PDF]

Supplementary Material

Frequency-specific adaptation and its underlying circuit model in the auditory midbrain

Li Shen, Lingyun Zhao, Bo Hong*

Department of Biomedical Engineering, School of Medicine, Tsinghua University, Beijing, China

*** Correspondence:** Bo HONG Ph.D., B204, Medical Sciences Building, Tsinghua University, Beijing, 100084, China.
hongbo@tsinghua.edu.cn

1. Supplementary Figures

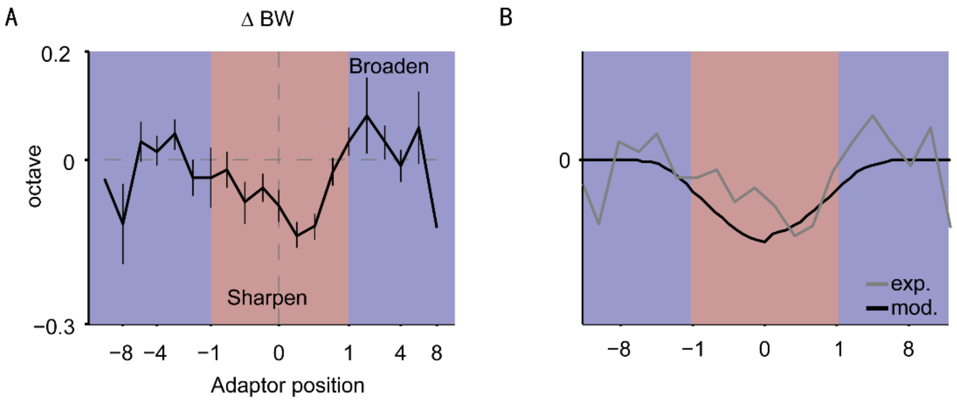

Supplementary Figure 1. Bandwidth change induced by different adaptors. (A) Bandwidth change of tuning curves varied as a function of adaptor positions (Mean ± SE). Center adaptors sharpen the tuning curves, while flank adaptors slightly widen the tuning curves. **(B)** The model with fitted parameters (fitted with narrowly-tuned neurons, black curve) shows a similar trend that center adaptors sharpen the tuning curves. When the adaptor was closer to the neuron's BF, it elicited larger magnitude of sharpening.

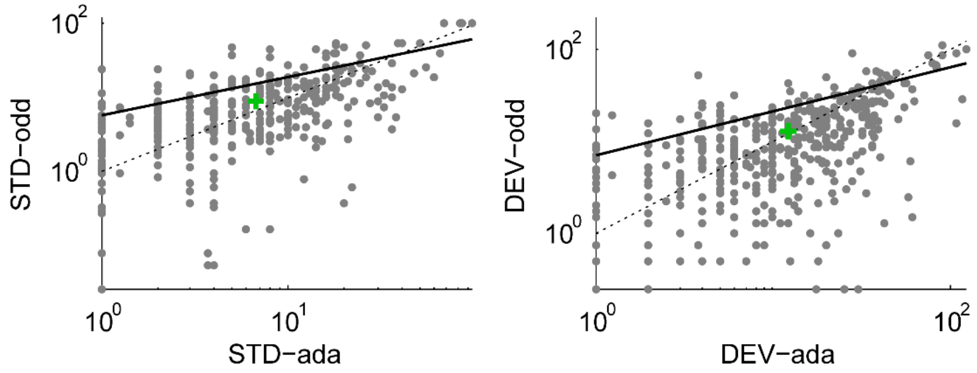

Supplementary Figure 2. The comparison of responses measured in biased ensemble with responses measured in classic oddball paradigm. (A) The comparison of the adaptor responses (STD-ada) with the standard responses in classic oddball paradigm (STD-odd). They are significantly correlated (Pearson's r = 0.47, p = 1.2×10-36). The black line shows the least square linear regression (Slope = 0.58). The dash line indicates a line with slope = 1. The green cross indicates the mean value of all the dots. **(B)** The comparison of the probe responses (DEV-ada) with the deviant responses in classic oddball paradigm (DEV-odd). They are significantly correlated (Pearson's r = 0.51, p = 7.9 ×10-45). Linear regression slope = 0.52.
